# Single Point Mutations Result in the Miss-Sorting of Glut4 to a Novel Membrane Compartment Associated with Stress Granule Proteins

**DOI:** 10.1371/journal.pone.0068516

**Published:** 2013-07-16

**Authors:** XiaoMei Song, Cheryl F. Lichti, R. Reid Townsend, Mike Mueckler

**Affiliations:** 1 Department of Cell Biology and Physiology, Washington University School of Medicine, St. Louis, Missouri, United States of America; 2 Department of Pharmacology & Toxicology, University of Texas, Galveston, Texas, United States of America; 3 Department of Medicine, Washington University School of Medicine, St. Louis, Missouri, United States of America; Cleveland Clinic Lerner Research Institute, United States of America

## Abstract

Insulin increases cellular glucose uptake and metabolism in the postprandial state by acutely stimulating the translocation of the Glut4 glucose transporter from intracellular membrane compartments to the cell surface in muscle and fat cells. The intracellular targeting of Glut4 is dictated by specific structural motifs within cytoplasmic domains of the transporter. We demonstrate that two leucine residues at the extreme C-terminus of Glut4 are critical components of a motif (IRM, insulin responsive motif) involved in the sorting of the transporter to insulin responsive vesicles in 3T3L1 adipocytes. Light microscopy, immunogold electron microscopy, subcellular fractionation, and sedimentation analysis indicate that mutations in the IRM cause the aberrant targeting of Glut4 to large dispersed membrane vesicles that are not insulin responsive. Proteomic characterization of rapidly and slowly sedimenting membrane vesicles (RSVs and SSVs) that were highly enriched by immunoadsorption for either wild-type Glut4 or an IRM mutant revealed that the major vesicle fraction containing the mutant transporter (IRM-RSVs) possessed a relatively small and highly distinct protein population that was enriched for proteins associated with stress granules. We suggest that the IRM is critical for an early step in the sorting of Glut4 to insulin-responsive subcellular membrane compartments and that IRM mutants are miss-targeted to relatively large, amorphous membrane vesicles that may be involved in a degradation pathway for miss-targeted or miss-folded proteins or represent a transitional membrane compartment that Glut4 traverses en route to insulin responsive storage compartments.

## Introduction

The rapid rise in circulating insulin levels after the ingestion of a carbohydrate-containing meal stimulates glucose transport into fat and muscle cells by causing the acute redistribution of the Glut4 glucose transporter from intracellular membrane storage compartments to the cell surface [1], [2], [3], [4], [5]. The resulting increase in glucose catabolism and storage in the form of glycogen and fat in these cells acts to buffer increases in blood glucose levels and prevent protracted postprandial hyperglycemia. A defect in the ability of Glut4 in muscle and fat cells to appropriately translocate to the cell surface in response to elevated circulating insulin levels is the proximal cause of peripheral insulin resistance [6], [7], a pathological state that is associated with obesity, metabolic syndrome, and type 2 diabetes mellitus [8], [9], [10], [11]. Consequently, much effort has been expended in an attempt to understand the molecular mechanism by which insulin regulates the subcellular trafficking of Glut4 and the possible derangements in this process that may result in insulin resistance.

The subcellular trafficking of Glut4 has been most thoroughly studied in cultured primary rat adipocytes and in the murine 3T3-L1 adipocyte cell line [12], [13], [14], [15]. Under steady-state basal conditions, i.e., in the absence of insulin, the bulk of Glut4 has been detected in several distinct intracellular membrane compartments, including endosomes, the trans-Golgi reticulum, and what appears to be a highly specialized membrane compartment that is usually referred to as Glut4 storage vesicles (GSVs) [16], [17], [18]. It is believed that in the basal state Glut4 moves among these intracellular compartments via vesicular-mediated budding and fusion events [18], [19]. Very little Glut4 can be detected in the adipocyte plasma membrane in the basal state [12], [13], [19], [20]. There is disagreement as to whether Glut4 recycles through the plasma membrane in the absence of insulin, and the extent to which recycling occurs may be dependent on the specific experimental conditions used, at least in 3T3-L1 adipocytes [14], [21], [22].

Although the precise subcellular itinerary that Glut4 follows after its biosynthesis in adipocytes remains poorly understood, it is generally agreed upon that very few other proteins share the intracellular trafficking of this molecule [23]. This implies that Glut4 possesses specific structural information that dictates its unusual insulin-regulated subcellular trafficking, and many studies have addressed this question over the past two decades [24], [25], [26], [27]. The complexity of Glut4 membrane trafficking suggests that several distinct structural targeting motifs are likely to be involved in this process, and experimental evidence is consistent with this assumption [28], [29], [30], [31], [32]. Several putative Glut4 trafficking motifs have been identified, including a di-leucine motif, a TELEY motif, and the IRM motif, all localized to the cytoplasmic carboxyl terminal domain [32], [33], [34], [35]. An FQQI targeting motif has been identified within the N-terminal cytoplasmic domain [36], [37], and undefined structural information within the central cytoplasmic loop of Glut4 appears to be crucial for the intracellular trafficking of newly synthesized molecules [38]. The specific phenotypes exhibited by mutations in these various putative trafficking motifs appear to vary depending on cell type and specific experimental conditions. A recent study in 3T3L1 adipocytes suggests that the FQQI and the TELEY motifs are involved in the intracellular retention of Glut4 in the basal state via recycling between endosomal compartments and the trans-Golgi network and GSVs, respectively [18], [39]. Mutation of either motif causes a partial redistribution of Glut4 to the cell surface and thus blunts the relative magnitude of insulin-induced redistribution to the cell surface. The di-leucine motif appears to be involved in the rapid internalization of Glut4 from the cell surface after insulin withdrawal [18].

Co-localization of wild type and mutant Glut4 molecules in individual cells under basal conditions is consistent with the roles proposed above for the 3 trafficking motifs. Mutant Glut4 molecules largely co-localize with wild-type Glut4, but in the case of mutations in the FQQI and/or TELEY motifs, an increase in cell surface localization is evident under basal conditions [18], [32]. In contrast, mutations in the IRM motif, which partially overlaps with the TELEY motif within the carboxy terminal cytoplasmic tail of Glut4, totally abolish insulin-stimulated translocation of Glut4 to the cell surface in addition to any detectable recycling of Glut4 through the plasma membrane in the basal state. The bulk of IRM mutant molecules are present in large dispersed cytoplasmic vesicles (LDVs) that lack wild type Glut4 [34].

In this study we further investigate the role of the IRM in Glut4 trafficking. We demonstrate that the two leucine resides within the IRM (L^500^ and L^503^) are critical for the appropriate subcellular trafficking of Glut4. Six different single or double point mutations involving either or both of these residues resulted in the mis-targeting of Glut4 similar to that observed for the original IRM mutant. In contrast to wild-type Glut4, The IRM mutant was heavily enriched in rapidly sedimenting vesicles after subcellular fractionation of 3T3L1 adipocytes. Immunogold electron microscopy indicated a high concentration of the IRM mutant in 150–250 nm vesicles that were closely associated with many unlabeled, smaller vesicles. Lastly, a sensitive proteomics analysis of immuno-enriched vesicles containing either wild type Glut4 or the IRM mutant demonstrated highly divergent protein compositions, suggesting that mutations in the IRM divert Glut4 to a “dead-end” trafficking pathway whereby Glut4 is unable to reach insulin-responsible membrane compartments.

## Materials and Methods

### Chemicals and Antibodies

DMEM and calf serum were purchased from Gibco. Fetal calf serum was purchased from ATLANTA biologicals (Lawrenceville, GA). BCA protein assay kits were purchased from Pierce (Rockford, Ll). RapiGest was from Waters (Milford, MA). Lipofectamin RNAiMAX Reagent, G3bp1 siRNA products “ggaacuuucuaugaucaga” and “agccuguaguggaaccaga”, G3bp2 siRNA “gaaugaugcgugaucguga” and “ggaaguuuaugcaaaccuu”, negative control#1 siRNA were from Life Technologies (Grand island, NY). All other chemicals from Sigma-Aldrich (St. Louis, MO). Polyclonal antibody against hemaglutinin (HA) was purchased from Abcam (Cambridge, MA). Monoclonal antibody against HA was purchased from Covance (Berkeley, CA). The Glut4 polycolonal antibody was described previously (Song et al.). Polyclonal antibody against Dsred was from Clontech (Mountain view, CA), Polyclonal antibody against G3bp1 was from Bethyl Laboratories (Montgomery, TX), Polyclonal antibody against G3bp2 was from LSBio (Seattle, WA). Rabbit IgG antibody was from Bethyl (Montgomery, TX). Colloidal Gold-affiniPure Goat anti-rat or mouse IgG were from Jackson ImmunoResearch (West Grove, PA).

### Cell Culture

3T3L1 fibroblasts (from American Tissue Type Culture Collection, Virginia) were grown in 20% calf serum in DMEM containing 25 mM glucose and supplemented with 10% glutamate, 1% penicillin, and 1% streptomycin. Two days after achieving confluence, the medium was changed to FBS medium (10% fetal bovine serum in DMEM supplemented with 10% glutamine and 1% penicillin and 1% streptomycin) with the addition of 670 nM insulin, 0.5 mM 3 isobutyl-methylxanthine, and 25 nM dexamethasome) for 2 days, and then replaced with FBS medium containing 670 nM insulin. After 48 hours, the differentiated cells were incubated in FBS medium, the cells were infected with adenovirus at day 5–6 overnight, and the adipocytes were then analyzed between days 8–10.

### Expression of Glut4 Constructs in 3T3-L1 Adipocytes

3T3-L1 adipocytes were infected with recombinant adenoviruses encoding Glut4 constructs as described in detail previously [34]. Large-scale viral stocks were routinely titered to determine the appropriate quantities required to equalize expression levels among the different constructs.

### Immunofluorescence Microscopy

3T3-L1 adipocytes were serum-starved for 2 hours, treated with or without 1 μM insulin for 30 minutes, washed with ice cold PBS buffer, and then fixed in 4% formaldehyde for 15 minutes at room temperature. The cover slips were washed with PBS for 15 minutes, incubated with primary anti-HA antibody or anti-G3bp1 antibody (Sigma-Aldrich, St. Louis, MO) and seconday antibodies (see ref 35). After mounting in Vectashield medium (Vector Laboratories, Inc. Burlinggame, CA) on glass slides, fluorescence images of the protein were then recorded using a ZEISS LSM-510 META laser confocal image system (Carl Zeiss, Thornwood, NY).

### Subcellular Fractionation and Sucrose Velocity Gradient Analysis

Adenovirus infected or uninfected 3T3-L1 adipocytes were serum starved overnight at day 8–9 post-differentiation. After washing 3x with cold PBS buffer, the cells were scraped into ice-cold HES buffer (50 mM Hepes, pH 7.4, 0.25M sucrose, 1 mM EDTA) containing a mixture of protease inhibitors (1 μg/ml leupeptin, 1 μg/ml benzamidine, 1 μg/ml chymostatin, 1 μg/ml pepstatin A, 5 μg/ml trypsin inhibitory, 0.082 units/ml aprotinin), and phosphatase inhibitor cocktail 2 (Sigma), homogenized, and then subjected to subcellular fractionation as described previously (Song et al, 2008). The SSV (formerly called LDM) and RSV (formerly called HDM) membrane fractions were resuspended in HES buffer and layered on top of a 10–30% linear sucrose gradient. The samples were centrifuged at 35,000 rpm for 1 hour at 4°C in a Beckman SW41 rotor, and 13 fractions were collected from the bottom of the tube. Equal amounts of the fractions were then subjected to SDS-PAGE.

### Immuno-isolation of Glut4 and IRM mutant membrane vesicles

Antibodies against Glut4 or Dsred were crosslinked to protein A Dynabeads (from Invitrogen, Carlsbad, CA) with 20 mM dimethylpimelimidate (Pierce) in 0.2 M triethanolamine (pH 8.2) for 30 minutes at room temperature. After quenching with 50 mM Tris (pH 7.5) for 15 minutes, the beads were washed with PBS. 1.5 mg of RSV or SSV protein from IRM/Glut4 adenovirus infected adipocytes or control adipocytes were resuspended in ice cold IC buffer (140 mM potassium glutamate, 20 mM Hepes, pH 7.4, 1 mM EGTA, 5 mM NaCl, 1 mM DTT) with protease inhibitors (1 μg/ml leupeptin, 1 μg/ml benzamidine, 1 μg/ml chymostatin, 1 μg/ml pepstatin A, 5 μg/ml trypsin inhibitory, 0.082 trypsin inhibitory units/ml aprotinin), and phosphatase inhibitor cocktail 2 (sigma), incubated with Dynabeads covalently conjugated to anti-Glut4 polyclonal antibody (for IRM/Glut4 expressing cells) or anti-rabbit IgG (for control cells) overnight at 4°C. The resulting supernatant was incubated with Dynabeads covalently conjugated with anti-Dsred polyclonal antibody (for IRM/Glut4 expressed cells) or rabbit IgG (for control cells) overnight at 4°C. The resulting beads were subsequently washed with IC buffer 5 times, and the proteins were then eluted with sample buffer containing 2% RapiGest, 100 mM Tris (PH 8.5), 1× reductant (Biorad, Hercules, CA).

### Protein Endoprotease Digestion and Peptide Preparation

The eluates from the bead immunoprecipitates were precipitated using the vendor protocol for the 2D clean-up kit (GE Healthcare, Pittsburgh, PA, Cat. No. 80–6484–51). The protein pellets were solubilized in 20 μl of Tris buffer (100 mM, pH 8.5) containing 8 M urea. The protein disulfide bonds were reduced with 1 mM TCEP (2 μl of a 50 mM solution) (TCEP bond breaker, 0.5 M solution, Thermo Fisher, Waltham, MA, Cat No. 77720) and placed at room temperature for 30 min. Alkylation of the cysteine residues was performed using iodoacetamide (2.2 μl of a 100 mM solution). After 30 min at room temperature in the dark, the reaction was quenched with 10 mM DTT at room temperature for 15 min. The reduced alkylated proteins (∼30 µl) were digested in 8 M urea with 1 µg of endoproteinase Lys-C (2 µl of a 0.5 µg/µl stock; Roche, Basel, Switzerland) after an overnight incubation at 37°C. The samples were diluted 1∶4 with 100 mM Tris, pH 8.5, trypsin (Sigma, Cat No. T6567) was added (∼1∶4 enzyme ratio), and the incubation was continued for 24 h at 37°C. The digests were acidified with aqueous 5% formic acid (3.3 μl) (Fluka, St. Louis, MO, Cat No. 56302). The peptides were extracted with a conditioned Nutip carbon tip (Glygen, Columbia, MD) (Cat No. NT3CAR). The tips were prepared by repetitive pipetting with 25 µl (×3) of the peptide elution solvent (60% acetonitrile in 1% formic acid and then equilibrated with 10 washes (25 µl) of extraction solvent (1% formic acid). The sample was loaded with 50 pipetting cycles. The tips were then washed four times with extraction solution. The peptides were recovered by 20 pipetting cycles with 25 µl of elution solution, followed by four washes (20 μl each) of elution solution. The extraction and wash solutions were combined in an autosampler vial (SunSri, Rockwood, TN, Cat No. 200 046) and dried in a Speed Vac (Thermo-Savant). The vial caps for the AS2 autosampler was from National Scientific (Rockwood, TN, Cat. No. 03–396AA).

### High-resolution Nano-LC-MS

Peptide mixtures were analyzed using high-resolution nano-LC-MS on a hybrid mass spectrometer consisting of a linear quadrupole ion-trap and an Orbitrap (LTQ-Orbitrap XL, Thermo Fisher Scientific). Chromatographic separations were performed using a nanoLC 2D Plus™ (Eksigent) for gradient delivery and a cHiPLC-nanoflex (Eksigent) equipped with a 15 cm ×75 μm C18 column (ChromXP C18-CL, 3 μm, 120 Å, Eksigent). The liquid chromatograph was interfaced to the mass spectrometer with a nanospray source (PicoView PV550; New Objective). Mobile phases were 1% FA in water (A) and 1% FA in acetonitrile (B). After equilibrating the column in 98% solvent A (aqueous 1% FA) and 2% of solvent B (acetonitrile containing 1% FA), the samples (5 µl) were injected from autosampler vials using the LC-system's autosampler at a flow rate of 500 nl/min followed by gradient elution (250 nl/min) with solvent B: isocratic at 2% B, 0–5 min; 2% B to 25% B, 5–110 min; 25% to 80%, 110–170 min; 80% to 2%, 170–175; and isocratic at 2% B, 175–190 min. Total run time, including column equilibration, sample loading, and analysis was 217 min. The maximum injection times for the MS1 scan in the Orbitrap and the LTQ were both 500 ms, and the maximum injection times for the MSn scan in the Orbitrap and the LTQ were 500 ms and 1000 ms respectively. The automatic gain control targets for the Orbitrap and the LTQ were 2×10^5^ and 3×10^4^ respectively, for the MS1 scans and for the MSn scan were 1×10^5^ and 1×10^4^ respectively. The MS1 scans were followed by six MS2 events in the linear ion trap with collision activation in the ion trap (parent threshold  = 1000; isolation width  = 2.0 Da; normalized collision energy  = 30%; activation Q  = 0.250; activation time  = 30 ms). Dynamic exclusion was used to remove selected precursor ions (−0.20/+1.0 Da) for 90 s after MS2 acquisition. A repeat count of 1, a repeat duration of 45 s, and a maximum exclusion list size of 500 was used. The following ion source parameters were used: capillary temperature 200°C, source voltage 4.0 Kv, source current 100 uA, and the tube lens at 110 V. The data were acquired using Xcalibur, version 2.0.7 (Thermo Fisher).

### MS Data Processing and Protein Quantification

**Figure 1:**
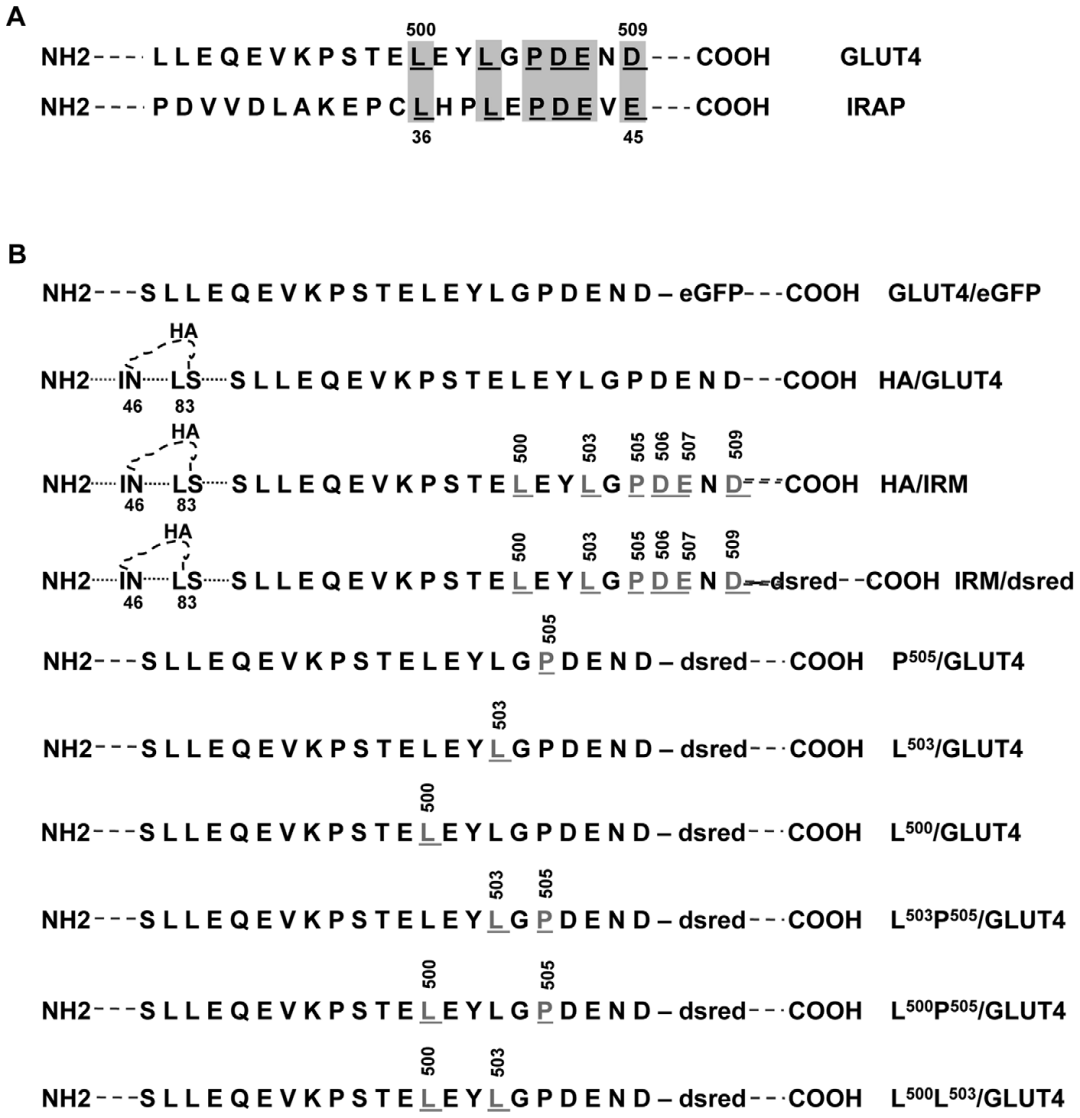
Diagrams of the IRAP and Glut4 sequence alignment used to identify the IRM mutant constructs. (A) Sequence alignment between the C-terminus of Glut4 and the N-terminus of IRAP; (B) HA/IRM and IRM/Dsred were tagged with the HA epitope within its first exofacial loop. Glut4/eGFP and all other mutants were tagged with GFP or Dsred at their C-termini. Amino acid residues are designated by the single-letter code. The gray and underlined letters are the amino acids that were mutated to alanine residues.

The LC-MS data processing pipeline is detailed in Figure S2. For protein identification, the LC-MS/MS files that were acquired using Xcalibur were processed using Mascot Distiller software (ver 2.0.3) for the preparation of files for database searching. A UNIPROT mouse protein database (downloaded May 2011, with 135387 sequences) with an added bovine serum albumin sequence (Uniprot accession No., P02769) was searched using Mascot software (ver. 2.2.04) with the parameters previously described (Morales DM et al (2011) Molecular and Cellular Proteomics (see Figure 1). The protein database searches were further processed using Scaffold software (ver. 3_00_07) and the proteins were identified using the Protein Prophet algorithm [40] with protein and peptide thresholds of 95% and 50%, respectively. The identified peptide sequences and mass spectrometric data that were used for protein identifications are given in Table S4.

For relative protein quantification, the LC-MS unprocessed files were imported into Rosetta Elucidator™ (Rosetta Biosoftware, ver 3.3) for retention time alignment of the peptide ion currents across the chromatographic time window using previously-described parameters [41] that are detailed in Figure S1. The aligned, normalized peptide ion currents were annotated within the alignment software by generating database search files (*.dta) and were annotated at the feature level of the software. By correlating the protein identifications with the Protein Prophet criteria used in Scaffold, as stated above. The ion current signals from all charge states for each peptide were concatenated unique using a visual script within the software. The table of peptides and peptide intensities was exported in Excel *.csv format. The peptides were grouped as individual genes. The gene-grouped and peptide intensity data were imported into DanTE-R for statistical analysis [42], [43].

### Deep-etch Electron Microscopy

Glass chips were cleaned with Chromic Sulfuric acid and ddH_2_O, carbon coated and glow discharged for 1 minute. After incubation in 0.1 mg/ml protein A for 30 minutes at room temperature and washing with PBS, the glass chips were blocked with 1% BSA in PBS for 30 minutes, coated with polyclonal anti-Glut4 or Dsred antibody or control anti-rabbit IgG for 1 hour at room temperature (see figure S2). The chips were incubated with SSV/RSV fraction or post Glut4 immuo-adsorbed RSV/SSV fraction overnight at 4°C. After washing with PBS, the vesicle-caoated glass chips were fixed with 2% formaldehyde in buffer containing 70 mM KCl, 30 mM Hepes, 5 mM MgCl_2_ (pH 7.2), 3 mM EGTA. After fixation for 15 minutes at room temperature, the chips were quenched with NaHCa buffer (100 mM NaCl, 30 mM Hepes, 2 mM CaCl_2_), plus 50 mM Lysine, 50 mM glycine and 50 mM NH_4_Cl for 30 minutes at room temperature. The chips were blocked with 1% BSA for 30 minutes, probed with IF8 mouse monoclonal or anti-rat Dsred or control IgG antibody for 30 minutes, washed with NaHCa buffer and then probed with 18 nm colloid gold-conjugated anti-mouse or 12 nm colloid-gold conjugated anti-rat IgG for 30 minutes. After washing with D_2_H_2_O, the samples were quickly frozen, freeze dried, and then replicated with platinum. Imaging was conducted using a “JEOL 1400 microscope”, and photographed with an AMT digital camera.

## Results

**Figure 2:**
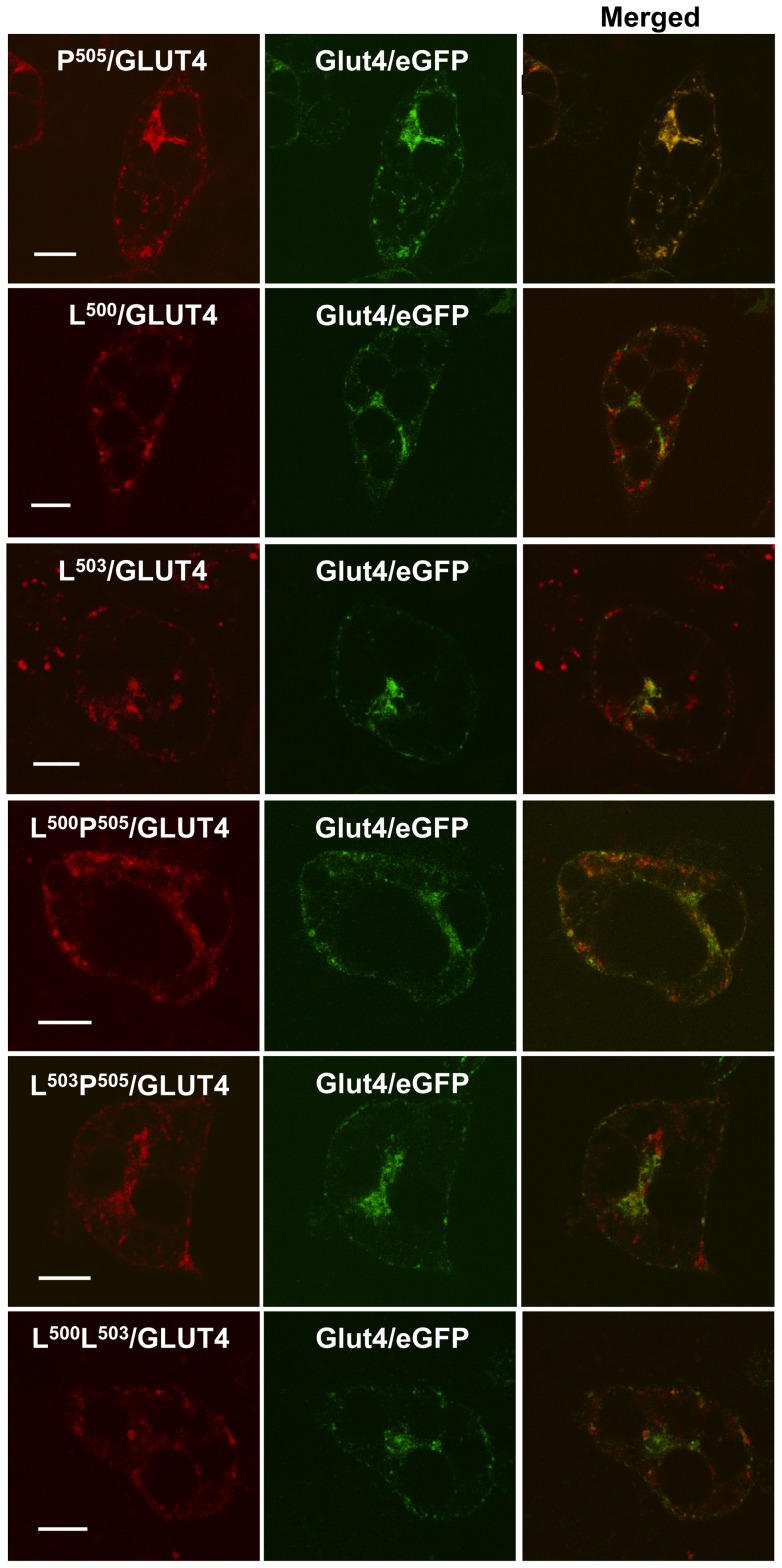
L^500^ and L^503^ are critical for the targeting of Glut4 to GSVs in the basal state. After co-expression of wild-type Glut4/eGFP and the various mutants within the IRM region of Glut4/Dsred by recombinant adenovirus infection, adipocytes were serum starved for 2 hours, washed with cold PBS, fixed with 4% paraformaldehyde, and then subjected to confocal microscopy analysis. The red color on the left panels represents mutants of Glut4/Dsred, the green color in the middle panels represents wild type Glut4/eGFP, and the yellow color in the right panels represents the colocalization of Glut4/eGFP and mutated Glut4/Dsred. The images were taken approximately through the middle of the cells and are representative of 4–5 independent experiments. The scale bar represents 10 μm.

**Figure 3:**
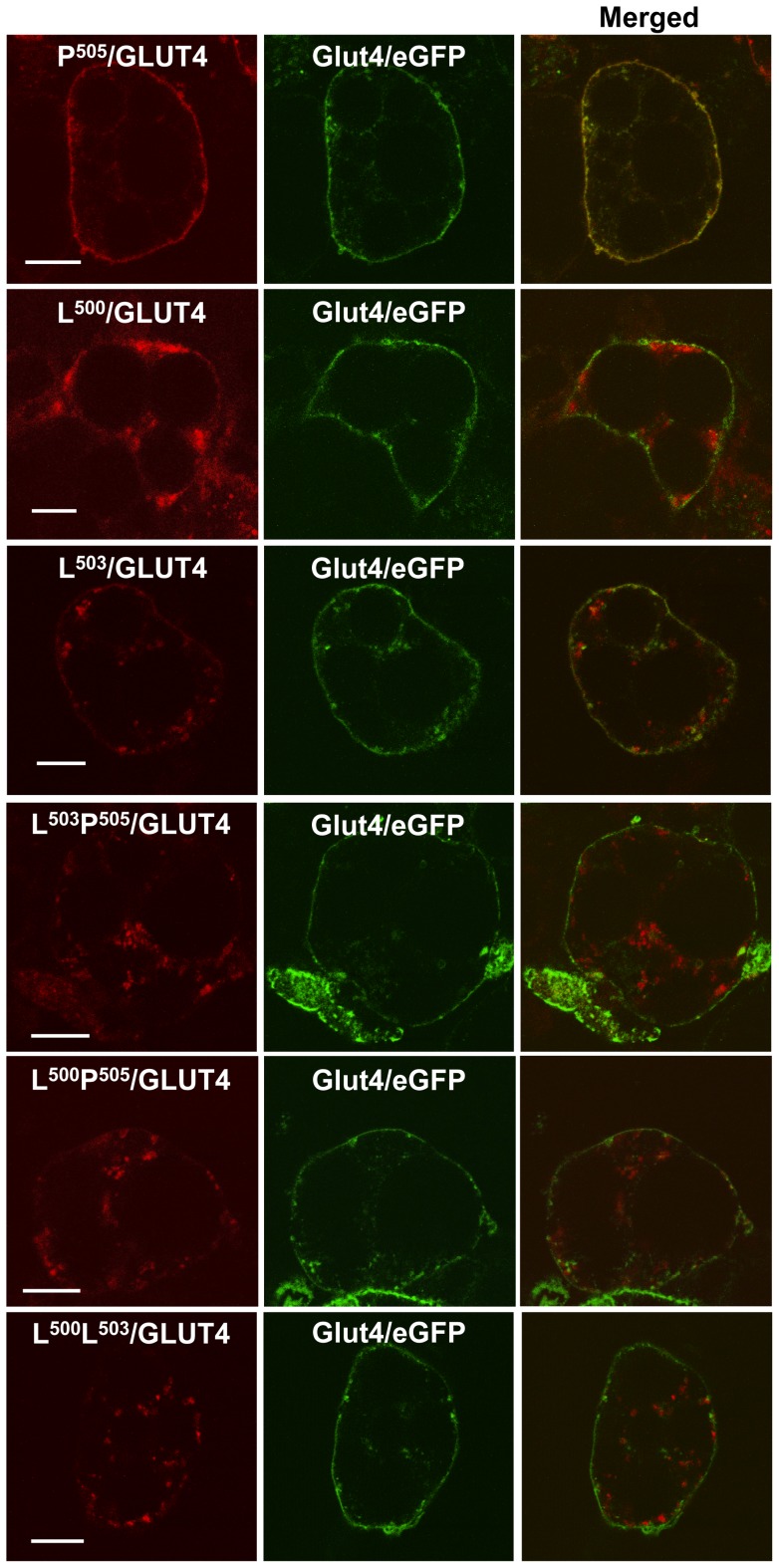
L^500^ and L^503^ are critical for insulin-stimulated translocation of Glut4 to the cell periphery. After co-expression of wild-type Glut4/eGFP and the various mutants within the IRM region of Glut4/Dsred using recombinant adenovirus infection, adipocytes were serum starved for 2 hours, stimulated with 1 μM insulin for 30 minutes, washed with cold PBS, fixed with 4% paraformaldehyde, and then subjected to confocal microscopy analysis. The red color in the left panels represents mutants of Glut4-Dsred, the green color in the middle panels represents wild type Glut4/eGFP, and the yellow color in the right panels represents the colocalization of Glut4/eGFP and mutated Glut4/Dsred. The images were taken approximately through the middle of the cells and are representative of 4–5 independent experiments. The scale bar represents 10 μm.

We previously noted the presence of sequence similarity between the extreme cytoplasmic C- terminal tail of Glut4 and a region within the amino terminus of the insulin responsive amino peptidase (IRAP), two membrane proteins that appear to share a major portion of their intracellular trafficking pathways in adipocytes (see Figure 1a). Two different sets of mutations within the IRM sequence motif (LXXLXPDEXD) were reported to result in aberrant subcellular targeting of Glut4 in the basal state and completely abolished its insulin-stimulated redistribution to the plasma membrane [34]. The possibility remained, however, that we had inadvertently introduced an unrelated and previously undefined dominant trafficking motif at the carboxy terminus of Glut4 in the two IRM mutants that were analyzed. We therefore examined the trafficking of 6 different single and double point mutations within the IRM sequence involving L^500^, L^503^, and P^505^ (see Figure 1b). The red fluorescent protein-tagged mutants were co-expressed in 3T3L1 adipocytes with green fluorescent protein-tagged wild type Glut4 in order to directly assess co-localization of the two different molecules within individual cells. We have previously demonstrated that wild type Glut4 targets indistinguishably whether it is tagged at its carboxyl-terminus with GFP or RFP, and that the tagged fusion proteins target very similarly to endogenous IRAP in 3T3L1 adipocytes [34]. Figure 2 shows that all 5 of the mutants in which either L^500^ and/or L^503^ were changed to alanine residues displayed aberrant targeting in the basal state compared to the wild type control, whereas the P^505^ mutant exhibited targeting that was indistinguishable from the wild type control under these conditions. Likewise, insulin-stimulated translocation of Glut4 to the cell periphery (representing presumptive plasma membrane insertion) was abolished in all 5 of the L^500^ and/or L^503^ mutants but remained intact in the single P^505^ point mutant (Figure 3). As observed previously, a much larger fraction of the aberrantly targeted mutants was present in large, dispersed, amorphous vesicles compared to the tagged wild-type Glut4, which was largely observed in small, diffuse, punctate structures and in a perinuclear compartment. Some co-localization between the mutants and wild-type transporters was observed in the perinuclear area.

**Figure 4:**
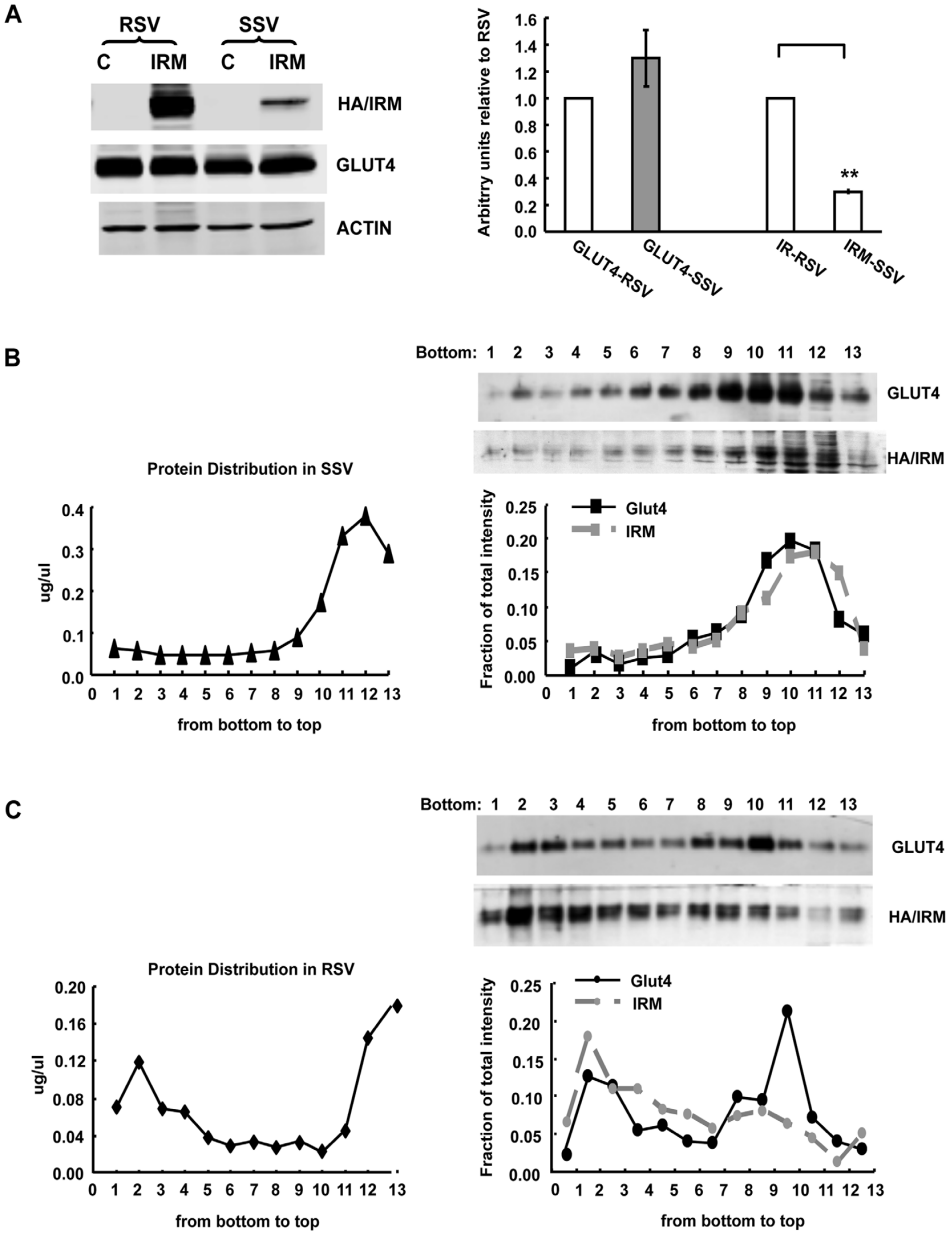
The IRM mutant was preferentially localized to the RSV fraction with a different sedimentation pattern. IRM/dsred mutant- (see Figure 1) infected adipocytes were serum starved overnight on day 8–9 post-differentiation and were then subjected to subcellular fractionation. The proteins from the RSV or SSV subcellular fractions were separated by SDS-PAGE, and then subjected to immunoblot analysis. (A). A representative western blot is shown on the left, and the quantification on the right shows the mean±SE from 3 independent experiments. “**” indicates P≤0.01 compared with control endogenous wild type Glut4. The isolated SSV (B) and RSV (C) subcellular fractions were subjected to sucrose velocity gradient analysis as described in “Experimental Procedures”. The fractions were collected from the bottom of the gradients and subjected to total protein quantification (left panels) or immunoblot analysis for endogenous wild-type Glut4 and the IRM mutant (upper pictures and lower quantifications). The data shown are representative of 2 independent experiments.

The difference in targeting between the IRM mutant and wild type Glut4 was also readily apparent after subcellular fractionation. A much higher fraction of mutant vesicles was present in the rapidly sedimenting vesicle (RSV) fraction on a per mg total protein basis versus the slowly sedimenting vesicle (SSV) fraction compared to endogenous wild type Glut4 (Figure 4A). Note that the IRM mutant is not recognized by the anti-Glut4 carboxy-terminal peptide antibody that was used to detect the endogenous wild type transporter. The SSV and RSV fractions were subjected to sucrose velocity gradient centrifugation and the distributions of endogenous wild-type Glut4 and the IRM mutant were determined across the gradients by quantitative immunoblot analysis. The IRM was detected using an antibody against the hemaglutinin tag placed in the first exofacial loop (see Figure 1). In the SSV fraction wild type Glut4 and the IRM mutant were both present in single peaks near the tops of the gradients, with the mutant peak displaced slightly nearer to the top of the gradient compared to wild type Glut4 (Figure 4B). In the RSV fraction wild type Glut4 was more heterogeneously distributed across the entire gradient but was largely present in two peaks representing smaller vesicles near the top of the gradient and larger vesicles near the bottom of the gradient (Figure 4C). This suggests that smaller membrane vesicles may have remained associated with larger vesicles during the initial differential centrifugation, but were separated or derived from the larger vesicles at some point before or during the linear sucrose gradient centrifugation step. Our unpublished data indicate that the small vesicles associated with the RSV fraction are not a non-specific sampling of trapped small vesicles from the SSV fraction, because they have a distinct protein composition (R. Hresko and M. Mueckler, unpublished observations). In contrast, the distribution of the IRM mutant in the RSV fraction was much more heavily weighted towards larger vesicles near the bottom of the gradient in comparison to wild type endogenous Glut4. These observations are consistent with the striking qualitative differences observed in the distribution of wild type Glut4 and the IRM mutant via confocal immunofluorescence microscopy [34](also see Figures 2 and 3), which showed that the IRM mutant is concentrated in large, dispersed, cytoplasmic vesicles under both basal and insulin-stimulated conditions.

**Figure 5:**
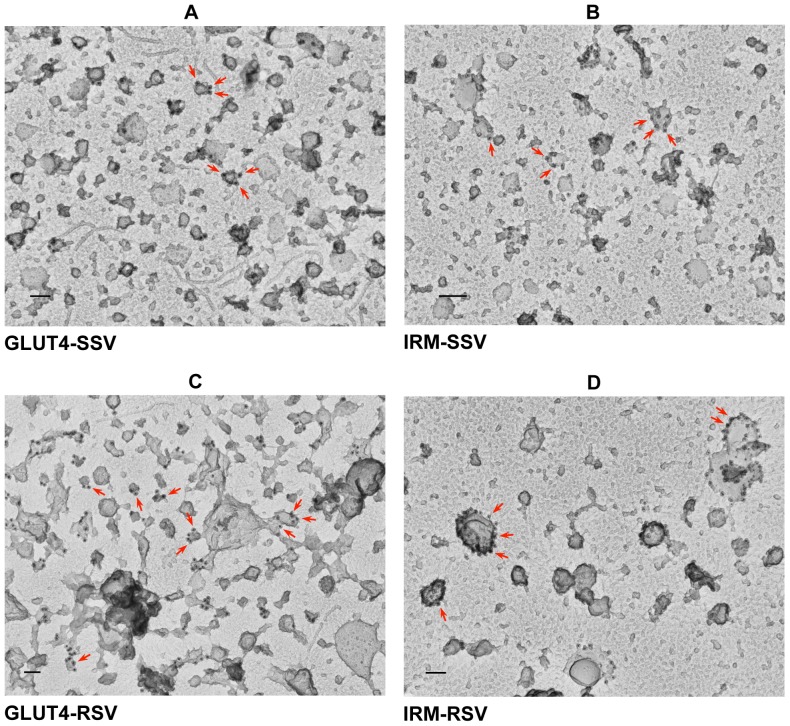
Immuno-gold electron microscopic localization of Glut4 and the IRM mutant in RSV and SSV vesicles. Control or IRM/dsred mutant (see Figure 1)-infected 3T3-L1 adipocytes were serum starved overnight. The cell lysates were subjected to differential centrifugation and the RSV and SSV fractions were used to immuno-adsorb endogenous Glut4-containing vesicles (A, C upper panel) or the exogenously expressed mutant IRM-containing vesicles (B, D) onto glass chips. The attached vesicles were labeled with immunogold-conjugated antibodies (18 nm gold conjugated anti-mouse IgG for Glut4 and 12 nm gold conjugated anti-rat IgG for the IRM mutant) and were then visualized by electron microscopy (see “Experimental Procedures”). The scale bars represent 100 nm.

SSV and RSV membrane fractions from 3T3L1 adipocytes expressing the Dsred/HA-tagged IRM mutant were immunoadsorbed onto glass chips coated with anti-Glut4 or anti-Dsred antibodies, subjected to secondary immunogold labeling after incubation with anti-Glut4 or anti-Dsred antibodies, and then visualized by deep etch electron microscopy. The SSV fractions contained small vesicles of relatively uniform size (<100 nm), many of which were labeled with gold particles directed against either the endogenous wild type Glut4 (Figure 5a) or the ectopically-expressed Dsred-tagged IRM mutant (Figure 5b). The Glut4-adsorbed RSV fraction contained a mixture of small (50–100 nm) free vesicles and larger vesicles (150–250 nm) that often were associated with branching structures and smaller vesicles (Figure 5c). Gold particles were observed on both small and large, branching structures in this fraction. In contrast, the Dsred-adsorbed RSV fraction contained a preponderance of larger vesicles (100–250 nm), many of which were densely labeled with gold particles directed against the IRM mutant (Figure 5d). These data are consistent with the observations obtained using light microscopy and subcellular fractionation. Interestingly, many of the gold particles in the Glut4-RSV fraction were associated with branching structures connected to larger vesicles, whereas most of the gold particles in the IRM-RSV fraction were directly associated with the bodies of large vesicles.

**Figure 6:**
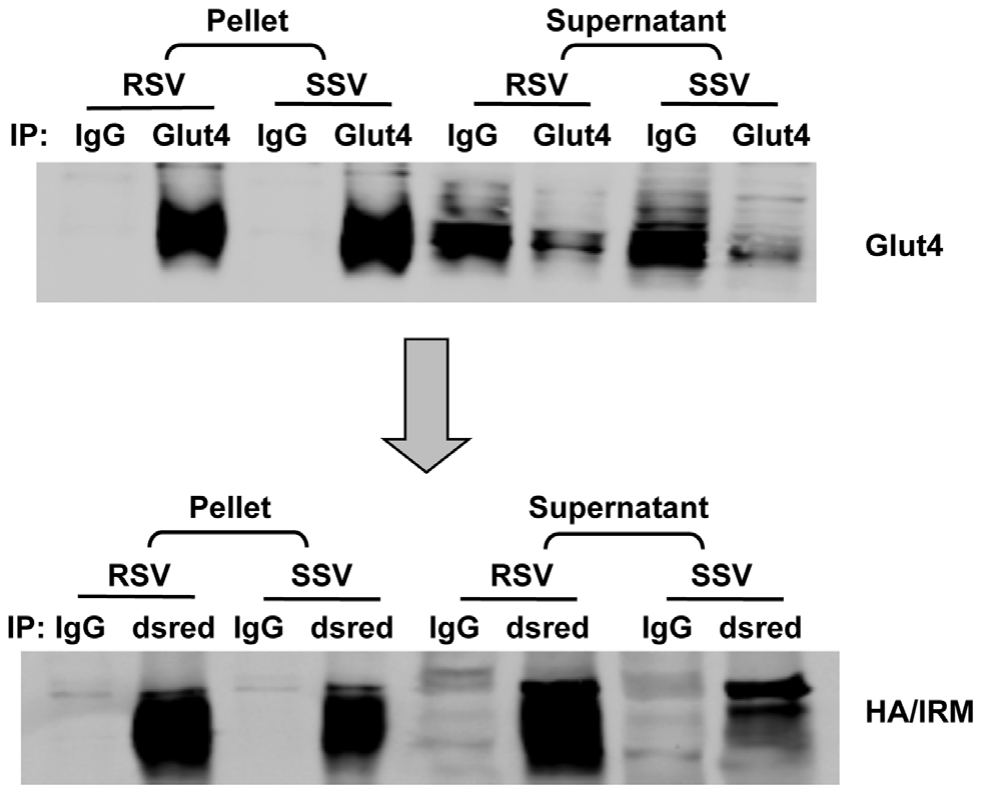
Immuno-adsorption of Glut4 and IRM mutant vesicles for Mass Spectrometry. RSV and SSV vesicles were pre-cleared with anti-rabbit IgG beads for 2 hours and the supernatant fractions were immuno-adsorbed with polyclonal anti-Glut4 magnetic beads. The supernatant fractions were subsequently subjected to immuno-adsorption with anti Dsred beads (to adsorb the mutant-containing vesicles). After washing, equal aliquots of the eluates from the beads were subjected to SDS PAGE and then subjected to immuno-blotting with monoclonal IF8 anti-Glut4 or anti-HA antibody (to detect the IRM mutant).

In a further effort to characterize the distinct membrane compartments to which the IRM mutant was routed, vesicles containing Glut4 or the IRM mutant were isolated from SSV and RSV fractions by immunoadsorption using a magnetic bead procedure. The magnetic bead procedure was critical to obtaining highly enriched vesicles from RSV, because standard centrifugation-based immunoadsorption procedures resulted in a large degree of non-specific sedimentation of the vesicles in this fraction (R. Hresko and M. Mueckler, unpublished observations). RSV and SSV fractions isolated from IRM mutant- expressing 3T3L1 adipocytes were first subjected to immuno-enrichment using beads coated with anti-Glut4 antibodies. This resulted in a>95% recovery of Glut4 vesicles from the membrane fraction and a>20-fold enrichment of Glut4 in the pellets (Figure 6). The supernatants from this initial immunoadsorption reaction were then cleared of >99% of Glut4 by two additional immunoadsoptions using anti-Glut4 magnetic beads. The supernatants obtained after the third anti-Glut4 immunoadsorptions were then used to immunoadsorb vesicles containing the Dsred-tagged IRM mutant. The recovery of the IRM mutant after immuno-enrichment (∼60%) was less efficient than that for Glut4 for reasons that are not evident.

The immuno-enriched Glut4 and IRM vesicles were then subjected to high resolution nanospray liquid chromatographic mass spectroscopy (nano-LC-MS) and the proteins present in each fraction were identified using the sequential scheme outlined in Figure S1. The results from two independent large-scale immuno-enrichment preparations and three independent nano-LC-MS analyses are summarized in Tables 1 and Table S1–3, which identify the proteins significantly enriched in each of the 4 different fractions (Glut4-SSV, Glut4-RSV, IRM-SSV, IRM-RSV) relative to control IgG fractions (P<0.05) for which a minimum of 2 distinct peptides were identified. The Glut4-SSV, Glut4-RSV, IRM-SSV, and IRM-RSV fractions contained 132, 127, 121, and 62 proteins, respectively, according to this analysis. The control IgG immunoadsorptions identify only those contaminant proteins that have a non-specific affinity for IgG. Consequently, many of these proteins, especially those that show a<2-fold degree of enrichment, are likely to be non-specific contaminants that are relatively abundant and/or have a high affinity for lipid/protein vesicles. This probably includes most of the proteins listed in the “Miscellaneous” category, especially those proteins that are known residents of mitochondria or the endoplasmic reticulum. As expected, Glut4 peptides were highly enriched in all 4 fractions with very high levels of statistical significance, confirming the efficiency of the immunoadsorption reactions (see Supplemental Table 1–3 and Tables 1). Note that although endogenous wild-type Glut4 was very efficiently cleared from the IRM mutant-enriched vesicle fractions, the ectopically expressed tagged IRM mutant shares peptides with the native wild type transporter that were identified by the nano-LC-MS analyses, and the IRM mutant does not react with the C-terminal Glut4 antibody.

**Table 1:** Proteins Identified in the IRM Mutant RSV Fraction from 3T3-L1 Adipocytes by Nano-LC-MS.

Primary protein name	Effect Size	P Value	Protein Description
**Membrane cargo proteins:**			
Slc2a4	5.86	1.75E-07	Solute carrier family 2, facilitated glucose transporter member 4
Scp2	3.04	4.51E-03	Non-specific lipid-transfer protein (EC 2.3.1.176) (NSL-TP) (Sterol carrier protein 2).
Atp6v1b2	2.44	6.00E-04	V-type proton ATPase subunit B, brain isoform
Igf2r	1.79	5.90E-03	Cation-independent mannose-6-phosphate receptor
Lrp1	1.75	1.49E-02	Prolow-density lipoprotein receptor-related protein 1
Anxa1	1.72	1.45E-04	Annexin A1
Anxa6	1.71	4.73E-09	Annexin A6
Anxa4	1.46	2.49E-02	Annexin A4
Atp5h	1.38	3.77E-03	ATP synthase, H+ transporting, mitochondrial F0 complex, subunit d (Fragment)
Slc25a5	1.27	3.48E-02	ADP/ATP translocase 2
**Membrane trafficking/sorting proteins**			
Bst2	3.46	1.27E-02	Bone marrow stromal antigen 2
Naca	2.92	4.91E-04	Nascent polypeptide-associated complex subunit alpha, muscle-specific form
Dctn1	1.57	3.84E-02	Dynactin subunit 1
Scamp3	1.48	1.89E-02	Secretory carrier-associated membrane protein 3
**Snares**			
Stx12	1.64	1.24E-02	Syntaxin-12
Vamp8	1.50	1.45E-02	Vesicle-associated membrane protein 8
**Kinase and phosphatase**			
Gapdh	3.05	2.63E-03	Glyceraldehyde-3-phosphate dehydrogenase
Pygb	1.64	1.95E-05	Glycogen phosphorylase, brain form
**Coated and adaptor**			
G3bp2	7.31	1.05E-05	Ras GTPase-activating protein-binding protein 2
G3bp1	6.68	1.09E-06	Ras GTPase-activating protein-binding protein 1
Cltc	1.46	1.04E-04	Clathrin heavy chain 1
Iqgap1	1.42	5.91E-03	Ras GTPase-activating-like protein IQGAP1
Mapksp1	1.37	1.90E-02	Mitogen-activated protein kinase scaffold protein 1
**Secreted**			
Alb	3.48	5.98E-06	Serum albumin
Aimp1	3.23	6.02E-04	Aminoacyl tRNA synthetase complex-interacting multifunctional protein 1
Anxa2	1.31	4.63E-02	Annexin A2
**Cytoskeletal**			
Sptan1	2.16	5.73E-03	Spectrin alpha chain, brain
Myh9	2.06	2.62E-03	Myosin-9
Vim	1.80	2.73E-05	Vimentin
Tuba1b	1.44	9.07E-03	Tubulin alpha-1B chain
Tubb5	1.19	2.40E-02	Tubulin beta-5 chain
**Miscellaneous**			
Caprin1	3.71	7.88E-06	Caprin-1
Nudt21	3.56	1.09E-02	Cleavage and polyadenylation specificity factor subunit 5
Mrps36	3.33	3.46E-02	28S ribosomal protein S36, mitochondrial
Pabpc4	2.65	1.16E-02	Poly A binding protein, cytoplasmic 4
Psmd7	2.62	3.05E-02	26S proteasome non-ATPase regulatory subunit 7
Bckdha	2.62	9.76E-05	2-oxoisovalerate dehydrogenase subunit alpha, mitochondrial,
Bckdhb	2.56	4.51E-03	2-oxoisovalerate dehydrogenase subunit beta, mitochondrial
Psmd11	2.27	4.50E-03	26S proteasome non-ATPase regulatory subunit 11
Eef1d	2.25	1.03E-05	Elongation factor 1-delta
Timm44	2.24	2.77E-04	Mitochondrial import inner membrane translocase subunit TIM44
Fubp3	2.23	4.46E-02	Far upstream element (FUSE) binding protein 3
Decr1	2.20	9.04E-05	2,4-dienoyl-CoA reductase, mitochondrial
Ogdh	2.13	3.48E-02	2-oxoglutarate dehydrogenase E1 component, mitochondrial
Hnrnpa2b1	2.04	3.44E-02	Heterogeneous nuclear ribonucleoproteins A2/B1
Hadhb	1.88	4.57E-02	Trifunctional enzyme subunit beta, mitochondrial
Dld	1.79	2.13E-02	Dihydrolipoyl dehydrogenase, mitochondrial
Hsp90ab1	1.76	8.36E-04	Heat shock protein HSP 90-beta
Hadha	1.73	3.10E-03	Trifunctional enzyme subunit alpha, mitochondrial
Dctn2	1.72	1.15E-03	Putative uncharacterized protein
Pabpc1	1.72	6.33E-03	Polyadenylate-binding protein 1
Hbb-y	1.71	1.75E-02	Hemoglobin subunit epsilon-Y2
Glul	1.68	2.06E-02	Glutamine synthetase
2700060E02Rik	1.66	2.16E-03	UPF0568 protein C14orf166 homolog
Fasn	1.56	4.47E-03	Fatty acid synthase
Lamp2	1.49	2.94E-03	Lysosome-associated membrane glycoprotein 2
Cct2	1.45	3.78E-02	T-complex protein 1 subunit beta
Gpd1	1.42	4.66E-02	Glycerol-3-phosphate dehydrogenase [NAD+], cytoplasmic
Anxa5	1.34	4.29E-02	Annexin A5 (Lipocortin V) (Calphobindin I) (CBP-I) (Placental anticoagulant protein I).
Arl8b	1.32	2.12E-02	ADP-ribosylation factor-like protein 8B
Mdh2	1.27	1.67E-02	Malate dehydrogenase, mitochondrial precursor (EC 1.1.1.37).

The “Effect Size” represents the values relative to the corresponding IgG control group, the “P Value” represents the statistical significance of enrichment compared to the corresponding IgG control groups.

**Figure 7:**
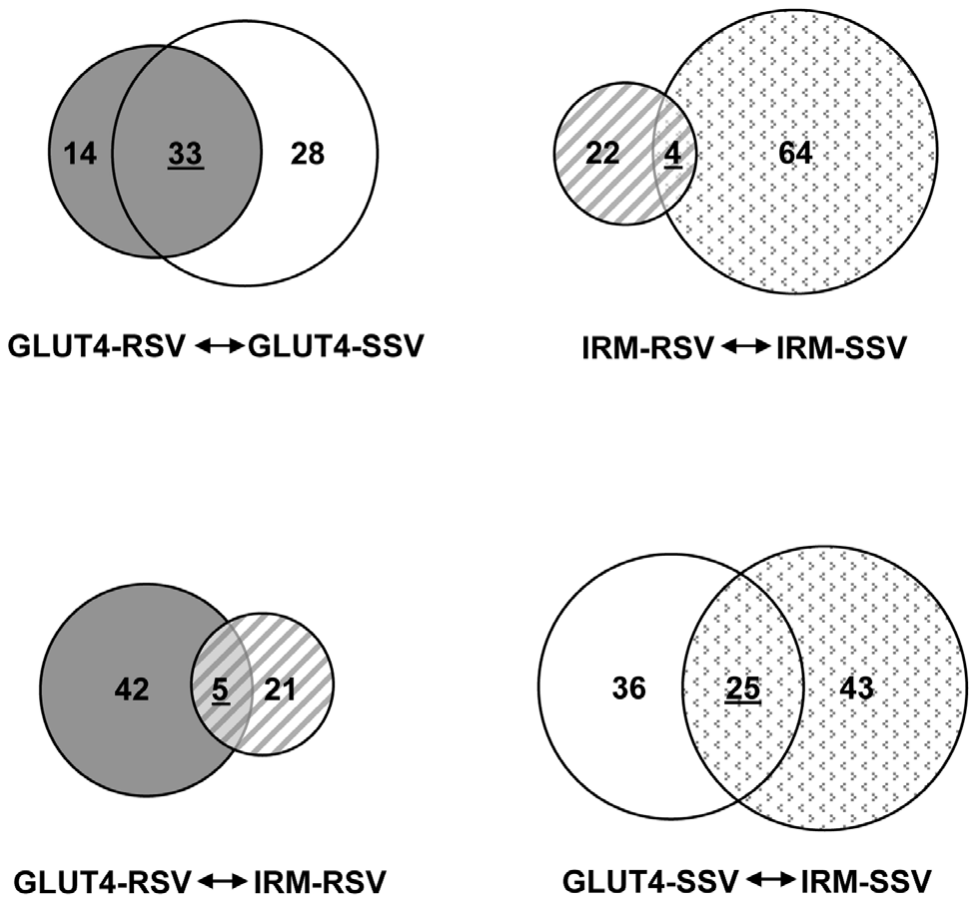
Diagram of the overlapping protein compositions of the Glut4 and mutant IRM enriched vesicle fractions according to nano-LC-MS analysis. Each number in the circle represents the unique proteins in that fraction relative to the other. The underlined numbers in overlapping areas between two circles represent the number of shared proteins between the two fractions. The gray circle represents Glut4-RSV, the blank circle represents Glut4-SSV, the small gray shaded circle represents IRM-RSV, and the gray dotted circle represents IRM-SSV.

Of the four vesicle fractions analyzed in the present study, only the Glut4 SSV fraction (traditionally referred to as the Glut4 low density microsomal (LDM) fraction) has been previously characterized by proteomic and immunological analyses. Our results confirm the presence of many proteins that have been reported to be present in the Glut4-SSV fraction by numerous studies [23], [44], [45] (proteins present in the Glut4-SSV fraction that have not been reported in previous studies are marked with an asterisk in Table S1). Among the best characterized of the known Glut4 vesicle proteins include sortilin, Glut1 (Slc2a1), mannose-6-P receptor, insulin- responsive aminopeptidase (Lnpep), transferrin receptor, IgF-II receptor, AS160 (TBC1D4), Vps45, carboxypeptidase D, Rab10, syntaxin16, syntaxin6, clathrin, and caveolin. Not surprisingly, most of these proteins are also present in the Glut4-RSV fraction (Table S2). The total RSV (“HDM”) fraction is enriched in markers for early biosynthetic compartments and probably contains compartments involved in the subcellular sorting of proteins associated with Glut4 intracellular vesicles. Of the 75 different proteins enriched at least 2-fold in either the Glut4-SSV or Glut4-RSV fractions, 33 (44%) of these were shared by the two compartments (see Figure 7).

Our previous studies demonstrated that the vast bulk of the IRM mutant was present in relatively large vesicular structures dispersed throughout the cytoplasm that lacked wild type Glut4 or any of several markers for various subcellular membrane compartments [34], suggesting that the mutant may be shunted to or trapped in a previously uncharacterized membrane compartment(s). The results shown in Figure 4 confirm the enrichment of the IRM mutant in relatively large vesicles. Interestingly, the IRM-SSV fraction, which contained only a small quantity of the total IRM mutant protein, shares a few of the well-characterized proteins present in the wild type Glut4-SSV fraction, including the mannose-6-P receptor, syntaxin16, syntaxin6, transferrin receptor, and AS160 (see Table S3). Of the 104 different proteins expressed in the Glut4-SSV and/or IRM-SSV fractions, 25 (24%) of these were shared by the two fractions (Figure 7). Strikingly, the IRM-RSV fraction, which contains most of the IRM mutant protein expressed in 3T3L1 adipocytes, contained only 26 distinct proteins that were enriched >2-fold relative to the IgG control (Table 1). A total of 90 different proteins were enriched >2-fold in either the IRM-RSV and/or IRM-SSV fractions, and only 4 of these (4.4%) were shared by both fractions. Only 5 of 68 proteins (7.3%) were shared between the Glut4-RSV and IRM-RSV fractions (Figure 7). Importantly, the most thoroughly characterized proteins present in Glut4 storage vesicles, i.e, IRAP, AS160, and sortilin, were not detected in the IRM-RSV fraction. The relatively small number of enriched proteins in the IRM-RSV fraction and the small degree of overlap in protein composition with the other three membrane fractions as a whole suggest that this subcellular fraction may primarily represent a single, distinct, membrane compartment.

**Figure 8:**
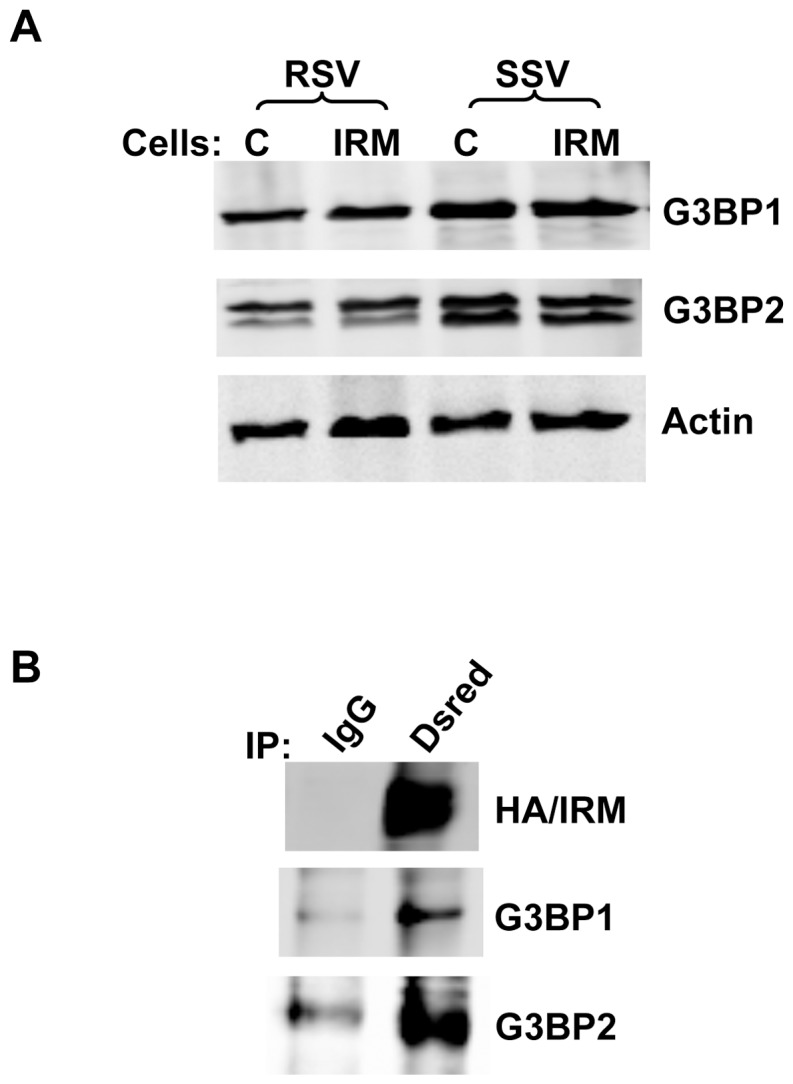
G3bp1/2 are present in immunoenriched IRM vesicles. (A) Cell lysates from control or IRM/dsred expressing adipocytes were subjected to differential centrifugation and the RSV and SSV fractions were subjected to immunoblot analysis using the antibodies indicated. (B) RSV fractions obtained as described above were subject to immunoadsorption with anti-Dsred or control IgG antibodies after pre-clearing endogenous Glut4 vesicles from the fractions using anti-Glut4 antibody. The eluates from the beads immuno-adsorbed with anti-IRM/dsred antibody or control IgG were analyzed by immunoblot analysis with the indicated antibodies.

**Figure 9:**
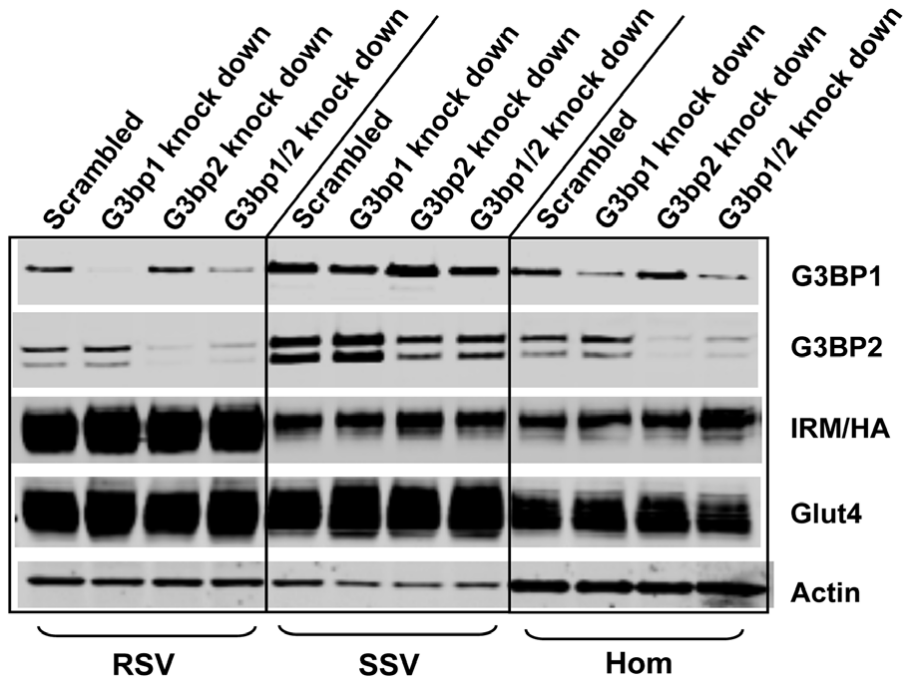
siRNA-mediated knockdown of G3bp1/2 expression does not affect levels of wild-type Glut4 or the IRM mutant in RSV and SSV fractions. Two days after infection with recombinant adenovirus expressing the IRM/dsred construct, the infected adippcytes and non-infected control adipocytes were transfected with siRNA directed against G3bp1 and/or G3bp2 mRNAs. After 2 h starvation, cell lysates from non-infected control adipocytes or IRM/dsred expressing adipocytes were subjected to differential centrifugation and the RSV and SSV fractions along with total cellular homogenates were subjected to immunoblot analysis using the antibodies indicated. Scrambled siRNA was used as a control. The blot shown is representative of 3–4 independent experiments.

Interestingly, the two proteins that have the greatest effect size in the IRM-RSV fraction, a parameter based on relative peptide intensities, are Ras GTPase binding proteins 1 and 2 (G3bp1 and G3bp2). G3bp1 and G3bp2 are proteins with multiple RNA and protein binding domains that are known to be involved in the formation of RNA stress granules [46], [47], [48]. They are also present in the cytosol and the nucleus but have not been previously reported on cytoplasmic membrane structures or studied in adipocytes [48], [49]. We therefore examined their possible role in the formation of the IRM vesicles in adipocytes. First, we confirmed the co-localization of G3bp1 and G3bp2 with purified IRM vesicles (Figure 8B). Next, we decreased expression of the proteins in adipocytes infected with recombinant adenovirus encoding the IRM mutant using siRNAs. Reducing G3bp1 and/or G3bp2 expression by 65–85% had no effect on the distribution of wild-type Glut4 or the IRM mutant between the RSV and SSV fractions and had no effect on the total expression levels of either of the Glut4 proteins (Figure 9). Since IRM expression did not alter the level of either G3bp1 or G3bp2 (Figures 8A), it appears that the IRM mutant either migrated to pre-existing vesicles enriched in G3bp1 or that IRM expression induced the partial migration of G3bp1/2 to a newly formed membrane compartment.

## Discussion

We previously reported the existence of a putative novel subcellular trafficking motif in the cytoplasmic carboxyl-terminal tail of the Glut4 glucose transporter (L^500^XXLXPDEXD^509^) (where X is any amino acid) [34]. The motif was identified by virtue of alignment with an identical sequence within the cytoplasmic amino terminal domain of IRAP, which is present in Glut4 storage vesicles and appears to share a very similar insulin-regulated subcellular trafficking pattern with Glut4 in adipocytes. Mutation of all 6 of the residues within this motif or of L^500^, L^503^ and p^505^ together to alanine residues resulted in the aberrant targeting of Glut4 in the basal state and totally abolished insulin-stimulated translocation of the transporter to the plasma membrane. Additionally, a fraction of intracellular wild type Glut4 was shown to recycle through the plasma membrane under basal conditions and then to equilibrate with the bulk pool of intracellular transporter molecules, whereas the IRM mutant completely failed to recycle in the presence or absence of insulin. This is by far the most dramatic phenotype ever reported for a Glut4 trafficking mutant, suggesting that the IRM plays a fundamental role, probably at an early stage, in the subcellular sorting of Glut4 molecules in 3T3L1 adipocytes. It is important to note that revealing the full extent of the aberrant phenotype of the IRM mutant was dependent on the co-localization of the mutant with a differentially tagged wild type Glut4 chimera co-expressed in the same cell population. Simply comparing different cell populations expressing either the wild type or IRM mutant would not have clearly demonstrated the extreme degree of miss-targeting of the mutant. Surprisingly, mutations in the IRM did not discernibly affect the trafficking or insulin responsiveness of IRAP. It is possible that IRAP “piggybacks” through the IRM sorting step on another molecule, but that begs the question as to why IRAP possesses the IRM sequence at all. Another possibility is that the IRM functions at a different step in IRAP trafficking and that mutations within the motif have a much more subtle phenotype for IRAP. The IRM appears to constitutively facilitate the trafficking of Glut4 to insulin responsive intracellular membrane compartments. There is no evidence that recognition of the IRM itself is regulated by the presence or absence of insulin. It should also be mentioned that it is not know whether mutations within the IRM affect transport activity of the protein, since IRM mutants are never inserted into the plasma membrane.

In the present study we examined the properties of the IRM mutant and the uncharacterized membrane compartments to which it is targeted. Both L^500^ and L^503^ are critical determinants of the IRM, since point mutations in either residue resulted in a phenotype very similar to that exhibited by the original IRM mutant. All 4 of the other residues within the IRM tolerate mutation to alanine residues, although this observation by itself does not negate the possibility that these residues do play a role in the recognition of the IRM by targeting factors. These data strongly suggest that mutations within the IRM do not result in the fortuitous creation of a dominant trafficking motif that results in the miss-targeting of Gut4, but that the IRM sequence comprises a motif that is critical for the eventual movement of the transporter into insulin responsive intracellular compartments. Because the IRM mutant never appears to reach the plasma membrane either in the presence or absence of insulin, it likely functions at a very early stage of Glut4 sorting, perhaps at the TGN. This hypothesis is also supported by data (X. Song and M. Mueckler, unpublished), which demonstrate that the IRM motif acts in a dominant fashion to the FQQI, di-leucine, or TELEY motifs when mutations in two of the motifs are introduced into the same Glut4 molecule.

The IRM mutant was much more highly enriched in the RSV fraction compared to wild type Glut4. When the RSV fraction was analyzed by sucrose velocity gradient centrifugation, wild-type Glut4 was present in two distinct peaks representing larger and smaller vesicle populations. Presumably the smaller vesicles associated with larger structures during the initial differential centrifugation, but separated from the larger vesicles during the velocity centrifugation step. In contrast, the IRM mutant was present in a single broad peak near the bottom of the gradients, representing a relatively large vesicle population. In the SSV fraction, which contained a relatively small proportion of the total IRM mutant, both endogenous wild type Glut4 and the mutant were present in single peaks representing small vesicles. These observations were confirmed and extended by immunogold labeling following by examination of the RSV and SSV fractions by deep etch electron microscopy. In the SSV fractions both wild type Glut4 and the IRM mutant were observed in 50–100 nm vesicles, and the abundance of vesicles was much lower on the grids coated with antibodies that recognized the IRM mutant. The Glut4-RSV fraction contained a mixture of small 50–100 nm vesicles and large (150–250 nm) vesicles, the latter often either associated with or consisting of branching structures. Interestingly, Glut4 was relatively abundant on the surface of the branching structures and was largely excluded from the bodies of the largest vesicles. Glut4 labeling was also observed in the small vesicles of the RSV fraction, which were abundant. In contrast, the IRM-RSV fraction lacked branching structures and was much more enriched in large vesicles (150–250 nm) compared to the Glut4-RSV fraction. IRM mutant gold labeling was highly concentrated on the surfaces of many of these large vesicles. It is tempting to speculate that the labeled branching structures in the Glut4-RSVs represent Glut4 moving through transitional membrane compartments, and that the large vesicles heavily labeled in IRM-RSVs represent the mutant present in a static, “dead-end” membrane compartment to which it is miss-targeted.

The 4 membrane fractions characterized in this study were analyzed by a highly sensitive nano-LC-MS procedure in order to define their protein compositions and characterize the compartments to which the IRM mutant was misdirected. The Glut4-SSV fraction (originally termed the “low-density microsomal fraction”) has been subjected to proteomic analyses in two previous studies [23], [44]. Our data confirm the presence of most of the proteins identified in these two studies (see Table S1), with a few notable exceptions. For example, the previously identified proteins, VAMP2, Rab4, and LRP1 were detected in the Glut4-SSV fraction in our analysis but are not listed in Table S1, because their enrichment above the controls did not quite achieve statistical significance. The previous proteomic studies did not report the statistical analysis of multiple MS runs. It should be noted that many if not most of the proteins identified by the MS analysis were probably associated non-specifically with the immunoadsorbed vesicles. For example, many resident mitochondrial proteins are listed in the tables that clearly cannot be specifically associated with Glut4 or IRM vesicles. The control fractions represent proteins that sediment with magnetic beads coated with non-specific IgG, and thus the control immunoprecipitates contain a small non-specific membrane vesicle content relative to the Glut4 and IRM mutant immuno-enriched fractions. We would therefore expect proteins that have a high affinity for membrane lipids per se to be enriched in the Glut4 and IRM vesicles fractions relative to the control fractions. Also, highly abundant proteins in general are more likely to be non-specifically trapped and detected in the larger Glut4 and IRM bead pellets. These are problems unique to the interpretation of proteomic analyses of immuno-enriched membrane vesicle populations that are well recognized and cannot be avoided.

Not surprisingly, 44% of the proteins in the Glut4-SSV fraction were also detected in the Glut4-RSV fraction (often referred to as the “high-density microsomal fraction”). These two vesicle fractions undoubtedly contain specific membrane compartments that are precursors to compartments in the other fraction, either during initial biosynthesis of Glut4 or during its steady state intracellular recycling, and thus it is expected that they would share many of the same proteins.

All of our data are consistent with the conclusion that only a very small proportion of the IRM mutant is present in small membrane vesicles. The IRM-SSV fraction shares 25 proteins with the Glut4-SSV fraction. This includes several proteins that are known to recycle, including the transferrin and mannose-6-P receptors [23], [50]; AS160, (TBC1D4) a protein known to be involved in the regulation of Glut4 trafficking [51]; and several proteins previously identified in Glut4 membrane compartments, including syntaxins 6 and 16, clathrin, and caveolin [33], [52], [53]. Interestingly, Cd36, a member of the class B family of scavenger receptors, is also shared between the Glut4-SSV and IRM-SSV fractions, but is much more highly enriched in the latter fraction. CD36 is involved in the binding and uptake of a diverse set of ligands, including long chain fatty acids, lipoproteins, collagen, phospholipids, and thrombospondin [54], [55]. The small proportion of the IRM mutant that appears in the SSV fraction may represent some leakage of the mutant into authentic Glut4 membrane compartments, but the majority of proteins in the Glut4-SSV and IRM-SSV fractions are not shared, suggesting that most of the small vesicles containing the IRM mutant represent one or more membrane compartments from which wild type Glut4 is excluded. For example, Glut1 (SLC2a1) is highly enriched in Glut4-SSV, but was not detected in IRM-SSV.

The vast bulk of the IRM mutant was detected in 150–250 nm vesicles in the IRM-RSV fraction. This fraction possessed the smallest number of proteins (26) that were >2-fold enriched of the IgG control and also shared the fewest number of proteins by far with the other fractions in pair wise comparisons (5 with Glut4-RSV and 4 with IRM-SSV). Of these shared proteins, two were shared with both Glut4-RSV and IRM-SSV (bone marrow stromal antigen 2 and the beta subunit of the V-type proton ATPase). These data strongly suggest that most of the IRM mutant molecules are present in one or more membrane compartments from which wild type Glut4 and other resident proteins of Glut4 vesicles are excluded.

The IRM-RSV fraction contains a heterogeneous mixture of membrane proteins and proteins that associate with membranes in various subcellular compartments. The 2 proteins that displayed the greatest enrichment relative to the non-specific IgG -coated beads were Ras GTPase-activating protein binding proteins 1 and 2 (G3bp1 and 2). Both G3bp-1 and G3bp2 are mRNA-binding proteins that are present in cytoplasmic stress granules that represent large clusters of mRNPS that are induced by various types of cellular stress and are not bounded by membranes [56], [57]. The precise role of stress granules and of G3pb in their formation is unclear. G3bps have also been associated with the regulation of signaling by the Ras family of GTPases and have been proposed to be involved in a variety of other cellular functions [46], [48], [58]. One possible explanation for the association of the IRM mutant with stress-granule associated proteins is that the mutant is funneled into a pathway by which misfolded or mis-targeted proteins are sequestered in a specific membrane compartment, associated with stress granules, for subsequent degradation. However, dozens of proteins have been identified in stress granules, and only 3 of these appear to be associated with the IRM compartment. An alternative explanation is that the localization of G3bp1/2 to the IRM compartment may reflect a currently unrecognized functional aspect of these proteins.

## Supporting Information

Figure S1 **Data processing for quantitative, label-free proteomics analysis of immunoprecipitates.** In step 1, the unprocessed LC-MS/MS files that were acquired using X-calibur (Thermofisher, ver. 2.0.7) were analyzed using Mascot Distiller software (ver 2.0.3) for preparation of files for database searching. After creating the *.mgf files, the MS2 data were searched using MASCOT (ver. 2.2.04) [41] against the UNIPROT mouse protein database (downloaded May 2011, with 135387 sequences) (Step 2). The MS1 and MS2 mass tolerances were set at 20 ppm and 0.8 Da, respectively. Carbamidomethyl was set as a fixed modification for Cys residues and Met residue oxidation was allowed as a variable modification. The protein database searches were further analyzed using Scaffold software (ver. 3_00_07) (Step 3) and the proteins were identified using the Protein Prophet algorithm [40] with protein and peptide thresholds of 95% and 50%, respectively (Step 4). The identified proteins and supporting mass spectrometric data are given in Table S1. For relative protein quantification, the same set of unprocessed LC-MS files were imported into Rosetta Elucidator™ (Rosetta Biosoftware, ver 3.3) and the peptide ion chromatograms were aligned and mean normalized using the following modification of the previously described parameters [59]): “Peak time score minimum  = 0.5; peak m/z score minimum  = 0.5; Scan width of m/z  = 350–1400; LC time range of 30–140 min; intensity scaling based on the mean intensity of all features (Step 5). The aligned peptide ion currents (PIC's) were annotated within the software by generating *.dta files (Step 6) and searching the UNIPROT human database using MASCOT as described above (Step 7). The ion current signals from all charge states for each peptide were concatenated unique using a visual script within the software. The table of peptides and peptide intensities was exported in Excel *.csv format (Step 8). In order to group peptide data generated from the products of each gene, the Mouse gene symbol was extracted from the UniProt database protein descriptor for each identified peptide. This was done using the following formula in Excel:  = LEFT(MID(AJ2, FIND(“GN = ”, AJ2)+LEN(“GN = ”),999), FIND(“PE = ”, MID(AJ2, FIND(“GN = ”, AJ2)+LEN(“GN = ”),999))–1) where AJ2 is the cell where the protein name exists. The spreadsheet was then sorted using Gene Symbol, to group them accordingly. The gene-grouped peptide intensity data were imported into DAnTE-R for statistical analysis [42], [43] (Step 8).
(TIF)

Figure S2 **Flow Chart of Deep-Etch Electron Microscopy.** Control or IRM mutant infected 3T3-L1 adipocytes were serum starved overnight. The cellular homogenates were subjected to subcellular fractionation and the RSV and SSV fractions were used for immuno-adsorption with control IgG or anti-Glut4 polyclonal antibodies attached to magnetic beads in order to clear the fractions by adsorption to non-specific IgG and/or to clear the fractions of vesicles containing wild-type Glut4. After glass chips were coated with either anti-Glut4 or anti-Dsred polyclonal antibody, the endogenous Glut4-containing vesicles or the exogenously expressed mutant IRM-containing vesicles were immuno-adsorbed onto the coated glass chips (4). The vesicles on the glass chips were fixed, probed with either mouse IF8 anti-Glut4 monoclonal (for endogenous Glut4) or rat anti-Dsred polyclonal antibody (for the IRM mutant), and then labeled with immunogold-conjugated secondary antibodies (18 nm gold conjugated anti-mouse IgG for Glut4 and 12 nm gold conjugated anti-rat IgG for the IRM mutant) and were then visualized by electron microscopy (see “Experimental Procedures”).
(TIF)

Table S1 **Identified proteins in Glut4 vesicles from SSV in 3T3-L1 adipocytes.** The “Log Effect” and “Effect Size” represent the value relative to the corresponding IgG control group, the “P Value” represents the significance of enrichment compared to corresponding IgG control groups.
(XLS)

Table S2 **Identified proteins in Glut4 vesicles from RSV in 3T3-L1 adipocytes.** The “Log Effect” and “Effect Size” represent the value relative to the corresponding IgG control group, the “P Value” represents the significance of enrichment compared to corresponding IgG control groups.
(XLS)

Table S3 **Identified proteins in IRM mutant vesicles from SSV in 3T3-L1 adipocytes.** The “Log Effect” and “Effect Size” represent the value relative to the corresponding IgG control group, the “P Value” represents the significance of enrichment compared to corresponding IgG control groups.
(XLS)

Table S4 **Mass Spectrometry and Database Search Results.** Database: the uniprot-mouse_20101228 database (Downloaded 5/2/2011, 135387 entries). Database searching: MASCOT DISTILLER version 2.3.0.0; MASCOT version 2.2.04. Protein Identification/Spectral Counting Quantification; Scaffold Proteome Software version 3.0.9.1.
(XLS)
