# Supplementary figures and images for: Single Point Mutations Result in the Miss-Sorting of Glut4 to a Novel Membrane Compartment Associated with Stress Granule Proteins
Source: PLoS One. 2013 Jul 16;8(7):e68516. doi: 10.1371/journal.pone.0068516 (PMC3713040; doi:10.1371/journal.pone.0068516)

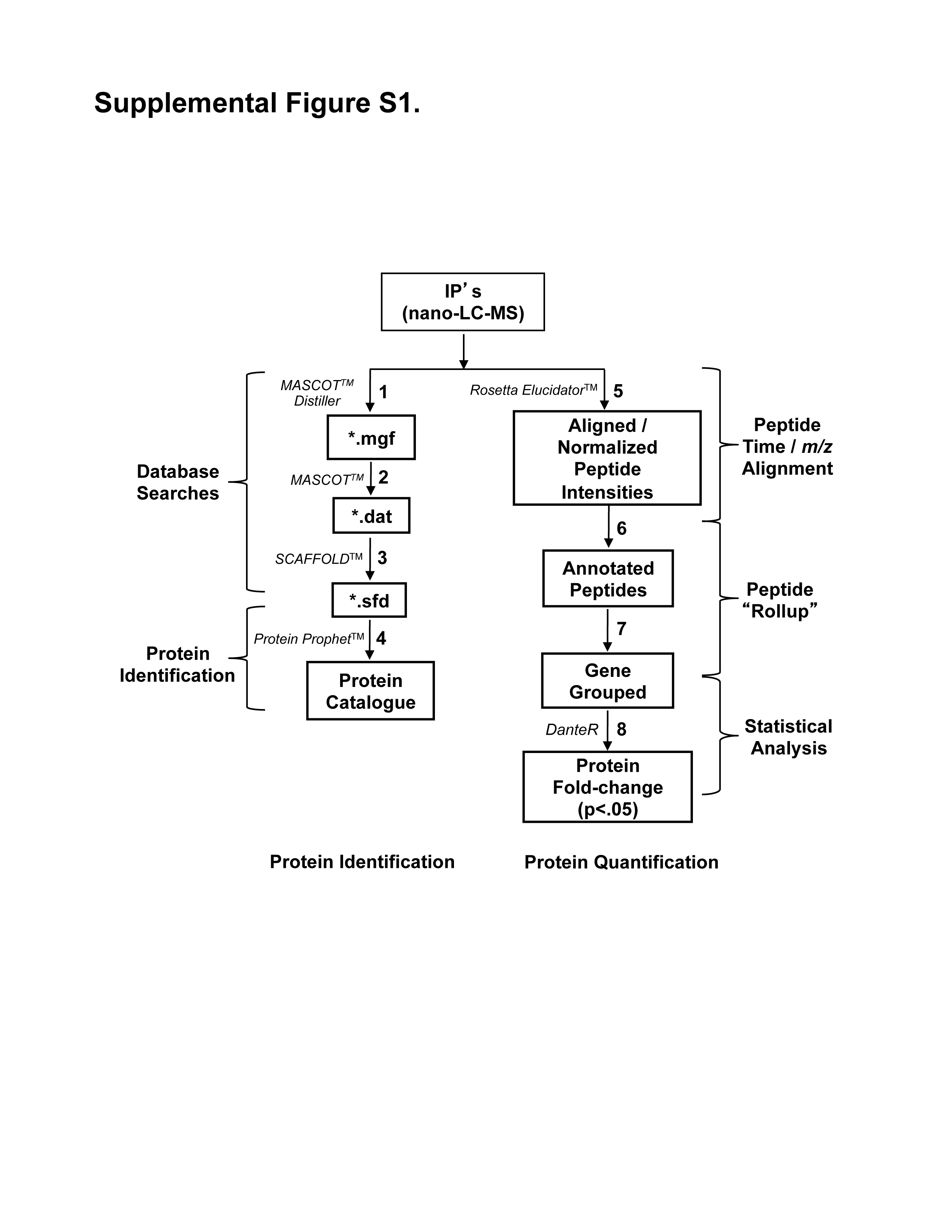

Supplement: Figure S1 — Data processing for quantitative, label-free proteomics analysis of immunoprecipitates. In step 1, the unprocessed LC-MS/MS files that were acquired using X-calibur (Thermofisher, ver. 2.0.7) were analyzed using Mascot Distiller software (ver 2.0.3) for preparation of files for database searching. After creating the *.mgf files, the MS2 data were searched using MASCOT (ver. 2.2.04) [41] against the UNIPROT mouse protein database (downloaded May 2011, with 135387 sequences) (Step 2). The MS1 and MS2 mass tolerances were set at 20 ppm and 0.8 Da, respectively. Carbamidomethyl was set as a fixed modification for Cys residues and Met residue oxidation was allowed as a variable modification. The protein database searches were further analyzed using Scaffold software (ver. 3_00_07) (Step 3) and the proteins were identified using the Protein Prophet algorithm [40] with protein and peptide thresholds of 95% and 50%, respectively (Step 4). The identified proteins and supporting mass spectrometric data are given in Table S1. For relative protein quantification, the same set of unprocessed LC-MS files were imported into Rosetta Elucidator™ (Rosetta Biosoftware, ver 3.3) and the peptide ion chromatograms were aligned and mean normalized using the following modification of the previously described parameters [59]): “Peak time score minimum = 0.5; peak m/z score minimum = 0.5; Scan width of m/z = 350–1400; LC time range of 30–140 min; intensity scaling based on the mean intensity of all features (Step 5). The aligned peptide ion currents (PIC's) were annotated within the software by generating *.dta files (Step 6) and searching the UNIPROT human database using MASCOT as described above (Step 7). The ion current signals from all charge states for each peptide were concatenated unique using a visual script within the software. The table of peptides and peptide intensities was exported in Excel *.csv format (Step 8). In order to group peptide data generated from the products of each [file pone.0068516.s001.tif]

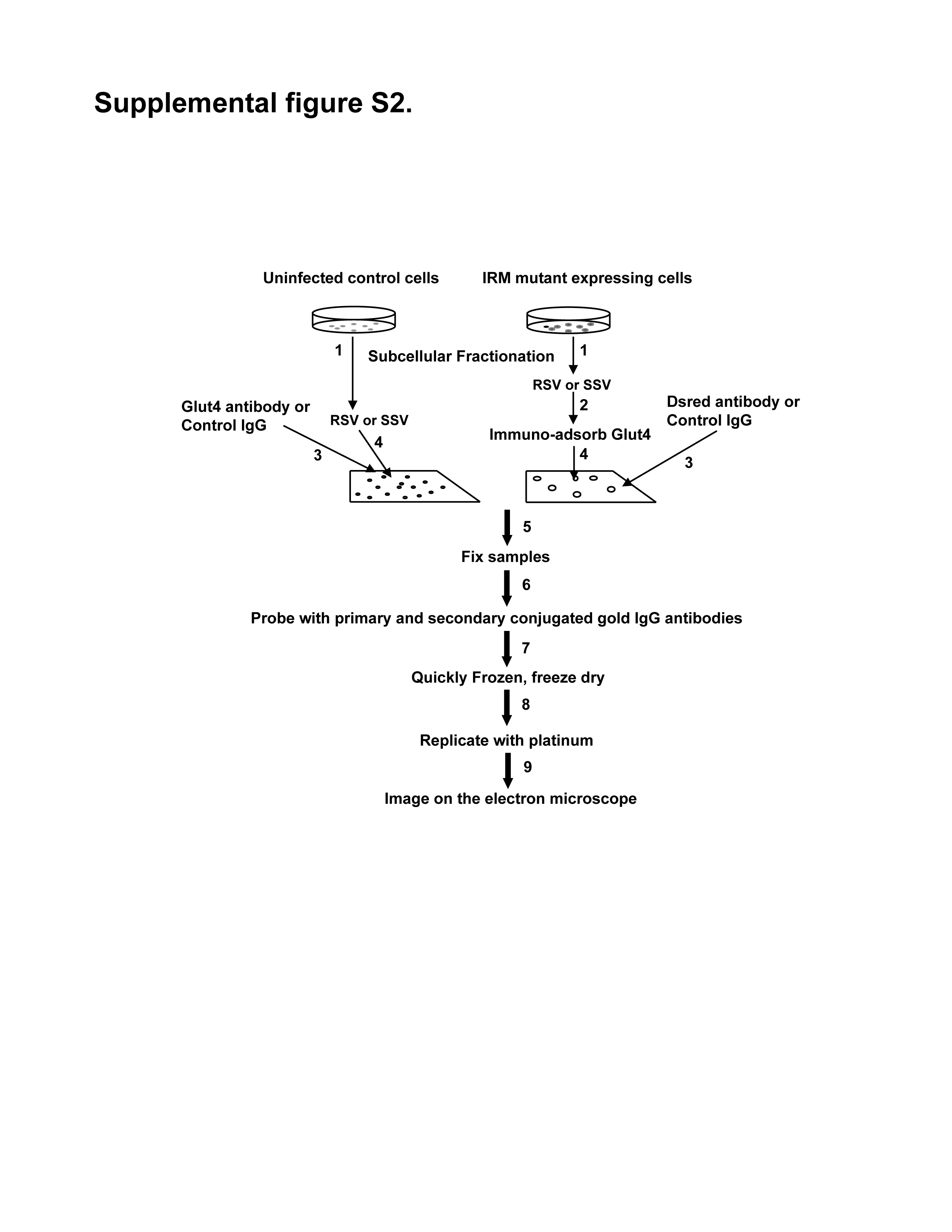

Supplement: Figure S2 — Flow Chart of Deep-Etch Electron Microscopy. Control or IRM mutant infected 3T3-L1 adipocytes were serum starved overnight. The cellular homogenates were subjected to subcellular fractionation and the RSV and SSV fractions were used for immuno-adsorption with control IgG or anti-Glut4 polyclonal antibodies attached to magnetic beads in order to clear the fractions by adsorption to non-specific IgG and/or to clear the fractions of vesicles containing wild-type Glut4. After glass chips were coated with either anti-Glut4 or anti-Dsred polyclonal antibody, the endogenous Glut4-containing vesicles or the exogenously expressed mutant IRM-containing vesicles were immuno-adsorbed onto the coated glass chips (4). The vesicles on the glass chips were fixed, probed with either mouse IF8 anti-Glut4 monoclonal (for endogenous Glut4) or rat anti-Dsred polyclonal antibody (for the IRM mutant), and then labeled with immunogold-conjugated secondary antibodies (18 nm gold conjugated anti-mouse IgG for Glut4 and 12 nm gold conjugated anti-rat IgG for the IRM mutant) and were then visualized by electron microscopy (see “Experimental Procedures”). (TIF) [file pone.0068516.s002.tif]
